# Scleroderma and scleroderma-like syndromes

**DOI:** 10.3389/fimmu.2024.1351675

**Published:** 2024-06-03

**Authors:** Katarzyna Romanowska-Próchnicka, Martyna Dziewit, Aleksandra Lesiak, Adam Reich, Marzena Olesińska

**Affiliations:** ^1^ Department of Connective Tissue Diseases, National Institute of Geriatrics, Rheumatology and Rehabilitation, Warsaw, Poland; ^2^ Departament of Dermatology, Pediatric Dermatology and Dermatological Oncology, Medical University of Lodz, Lodz, Poland; ^3^ Department of Dermatology, Institute of Medical Sciences, Medical College of Rzeszów University, Rzeszów, Poland

**Keywords:** systemic sclerosis, morphea, scleroderma, scleroderma-like syndromes, syndromes of inflammatory/autoimmune, genetic, toxic

## Abstract

Systemic sclerosis is a systemic connective tissue disease whose main pathophysiological mechanism is a progressive fibrosis of internal organs and skin leading to thickening and induration. Blood vessels may also be involved. However, systemic scleroderma is not the only disease causing cutaneous sclerosis. There is a group of diseases that mimic scleroderma in their clinical presentation - these are scleroderma-like syndromes. A distinction can be made between syndromes of inflammatory/autoimmune, genetic, metabolic, toxic, drug-induced, occupational, paraneoplastic and syndromes caused by deposition disorders. In the following paper, we have reviewed the literature on scleroderma-like syndromes. We have outlined the factors predisposing to the development of each disease, its pathogenesis, clinical presentation, diagnostic and treatment process and the differences between each syndrome and systemic scleroderma.

## 1 Introduction

**Figure 1:**
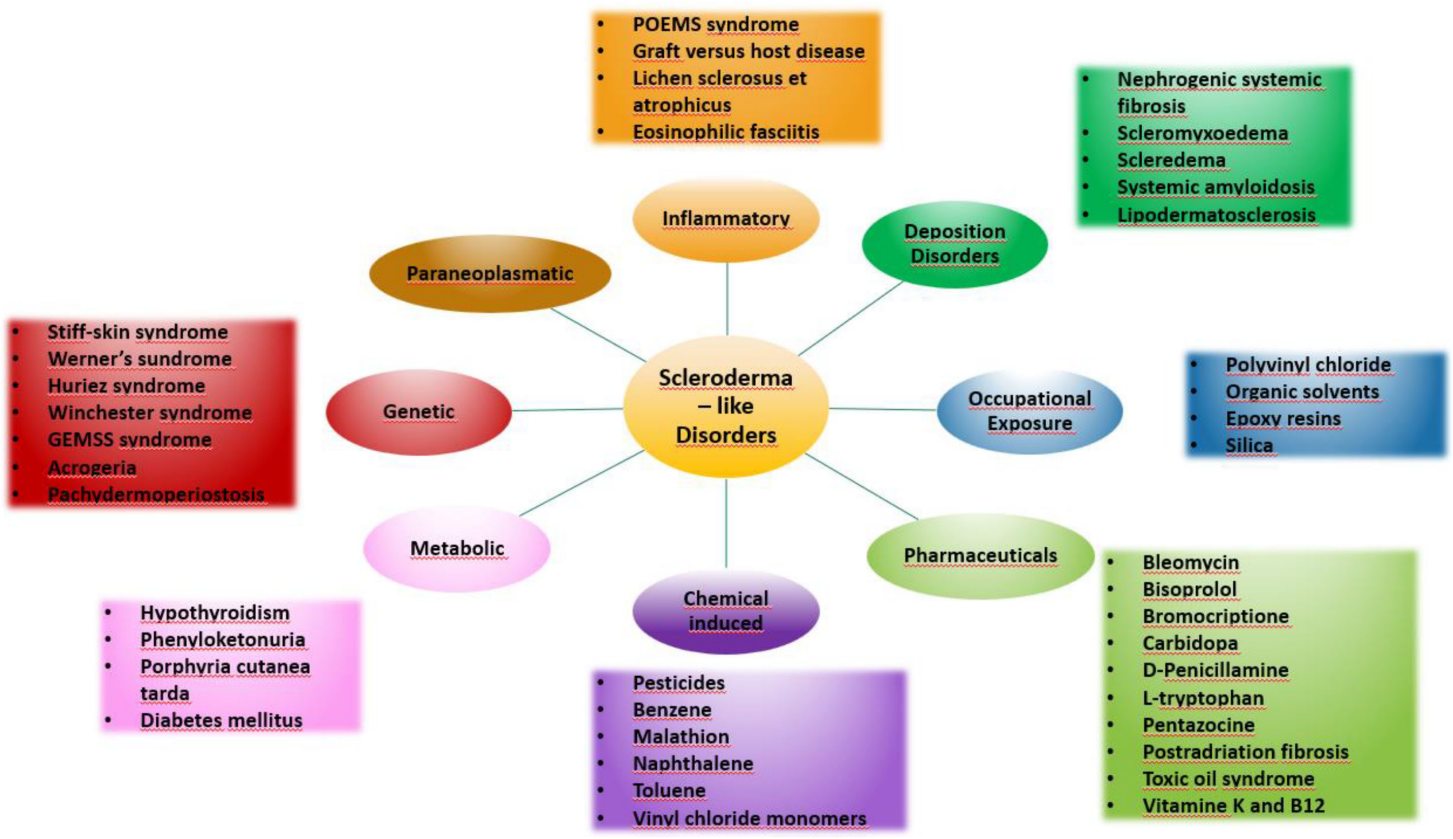
Scleroderma-like disorders.

**Figure 2:**
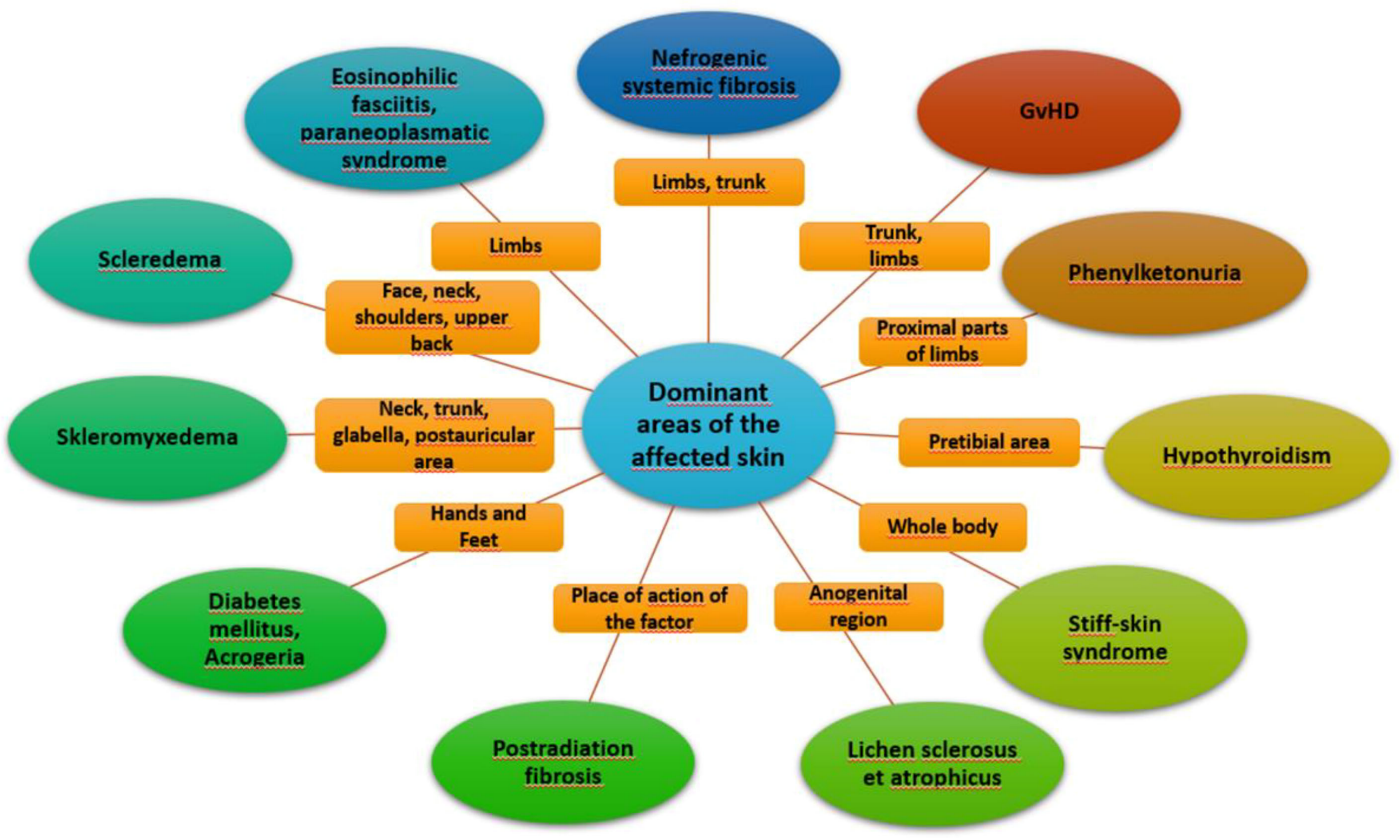
Dominant areas of affected skin.

Systemic sclerosis is a systemic connective tissue disease whose main pathophysiological mechanism is a progressive fibrosis of internal organs and skin leading to thickening and induration. Vascular involvement may also occur. Scleroderma-like syndromes are conditions that clinically resemble scleroderma, but differ in etiology, pathogenesis, treatment and prognosis. Scleroderma-like syndromes can be divided by the predominant involvement of a particular skin area or by their etiology - inflammatory/autoimmune, genetic, metabolic, toxic, drug-induced, occupational exposure, paraneoplastic, deposition disorders. The division is illustrated in **Figures 1**, **2**.

The knowledge of scleroderma-like syndromes, their triggering factors, symptoms and abnormalities in ancillary examinations will greatly help the clinician to differentiate between the individual conditions and systemic sclerosis and to initiate effective treatment. The main features that should be considered during the diagnosis are the characteristics of skin lesions, their distribution, the presence of Raynaud’s phenomenon, microangiopathy typical of scleroderma observed on capillaroscopy, the presence of ANA antibodies and the histopathological picture of biopsy specimens taken from the lesion area. **Table 1** compares scleroderma-like syndromes with systemic sclerosisbased on the above-mentioned features.

**Table 1:** Comparison of scleroderma and scleroderma – like syndromes.

DISORDER	ETIOLOGY	SKIN	DISTRIBUTION	SYSTEMIC CHANGES	LABORATORY	NAILFOLD CAPILLARIES and RAYNAUD'S PHENOMENON	TREATMENT
Scleroderma	Unknown	Thick, shiny, indurated, tight, skin ulcerations	Face, hands commonly, middle back spared	Dysphagia, interstitial lung disease, pulmonary arterial hypertension,	ANA, anti- centromere, anti- topoisomerasel, anti-RNA polymerase III:	Abnormal; present	MTX, CP. hydroxychloroquin e. MMF, rare systemic steroids
Eosinophilic Fascuitis	Unknown	Woody induration, groove sign, prayer sign, peau d'orange	Limbs. spares face	Carpal tunnel syndrome, joint contractures	Eosinophilia, hypergammaglobul inemia, elevated aldolase	Mostly normal; uncommon	Systemic steroids, MTX. CP
Lichen sclerosus et atrophicus	Unknown	Atrophic skin, keyhole sign, porcelain white papules, plaques, follicular delling and hyperkeratosis, hypopigmentation	Anogenital region	None	None	Normal, absent	TCS, clobetasol propionate 0.05%
POEMS syndorme	Unknown	Thick, hyperpigmentation hemangioma, hypertrichosis	Hands, feet, diffuse	Peripheral neuropathy, osteosclerotic bone lesion, endocrinopathy, multiple myeloma	IgG or IgA monoclonal gammopathy	Normal; present	Autologous stem cell transplantation, systemic steroids, melphalan, thalidomide
GvHD	Donor T- lymphocytes	Thick, indurated, lichen planus-like, , erythemous hypo- hyperpigmented plaques, skin ulcerations	Trunk, limbs	Myositis, gastrointestinal, liver, lung and lymphoid tissue changes	Anti-Scl, anti-PM- Scl, aPL, ANCA	Can be abnormal; absent	Systemic steroids, calicineurin inhibitors, extracorporeal photopheresis, thalidomide, rituximab
Nephrogenic systemic fibrosis	Gadolinium exposure	Indurated, nodular woody plaques, hiperpigmentation	Trunk, limbs; hands, feet and face spared	Renal failure, joint contractures, heart and lung fibrosis, peripheral neuropathy	Elevated serum creatinine	Normal; absent	Plasmapheresis, interferon, IVIG
Scleromyxedema	Unknown	Cobblestone indurated, waxy papules in linear arrays, plaques, "leonine" face. "Shar-Pei sign"	Glabella, posterior auricular area, neck, trunk	Dysphagia, pulmonary hypertension, arthralgia, polyarthritis, myopathy, seizures, encephalopathy, psychosis, coma	Monoclonal gammopathy (IgG lambda), paraproteinemia	Normal; uncommon	Melphalan, systemic steroids, plasmapheresis, retinoids, PUVA, IVIG, autologous stem cell transplantation
Scleredema	Unknown	Doughy, indurated	Face, neck, shoulders, upper back. spares hands and feet	Dysphagia, ocular palsy, hepatosplenomegal y, cardiomyopathy, pericardial effusion	Monoclonal gammopathy (IgG kappa), hyperglycemia	Normal; absent	Systemic steroids, PUVA, MTX, CsA, physical therapy
Porphyria cutanea tarda	Uroporphyrinogen decarboxylase defect	Bullae, ulcerations, hyperpigmentation, hypertrichosis, blisters, thick	Diffuse, areas exposed to sunlight	Liver damage	High level of porphyrin in urine and plasma	Normal; absent	Avoid of triggering factors, phlebotomy, chloroquine and hydroxychloroquin e
Phenylketonuria	Phenylalanine hydroxylase deficiency	Thick, indurated	Proximal parts of limbs, spare hands and feet	CNS involvement - microcephlay, seizure	High level of phenylalanine metabolites in blood, decrease level of tyrosine in the blood	Normal; absent	Low phenylalanine diet
Diabetic chieroarthropathy	Long-standing, uncontrolled DM	Prayer sign, thick	Hands, feet	Joint contractures, cardiovascular, renal, neurological changes	ICA. IAA, anti- GAD, hyperglycemia	Absent	DM treatment
Paraneoplasmatic syndrome	Serotonin and other mediators	Thick	Face, chest, limbs	Endocardial fibrosis	Increased daily excretion of 5- HIAA	No information	Surgical removal of tumor, octreotide

ANA, antinuclear antibodies; ANCA, antineutrophil cytoplasmic antibodies; aPL, antiphospholipid antibodies; CNS, central nervous system; CP, cyclophosphamide; CsA, cyclosporine; DM, Diabetes mellitus; GAD, Glutamic acid decarboxylase; IAA, Insulin autoantibodies; ICA, Islet cell autoantibodies; IgG, Immunoglobulin G; IVIG, intravenous immunoglobulins; MMF, mycophenolate mofetil; MTX, methotrexate; PUVA, Psoralen + ultraviolet light A; TCS, Topical corticosteroids.

In the following paper, we have reviewed the literature on scleroderma-like syndromes. We have outlined the factors predisposing to the development of each disease, its pathogenesis, clinical presentation, diagnostic and treatment process and the differences between each syndrome and systemic sclerosis.

Our main aim is to expand the knowledge of scleroderma-like syndromes and present the importance of taking them into account during the differential diagnosis of systemic sclerosis.

## 2 Systemic sclerosis

Systemic sclerosis is a rare systemic connective tissue disease with progressive fibrosis of the skin and internal organs, as well as microvascular damage and dysregulation of both adaptive and innate immunity (1). The most commonly affected organs include the skin, lungs, pericardium, kidneys, skeletal muscle and gastrointestinal tract. The disease is more common in women (F:M 5:1), more severe with late onset of first symptoms, in men and in African-Americans.

### 2.1 Systemic sclerosis is divided into

#### 2.1.1 Limited cutaneous systemic sclerosis

previously known as CREST syndrome. Cutaneous sclerosis involves the face and distal parts of the upper and lower extremities. Telangiectasia, sclerodactyly and calcinosis cutis maybe be notable, while scleroderma renal crisis and severe interstitial lung disease are uncommon. Raynaud phenomenon might precede other symptoms. Late stage complications are prevalent, especially gastrointestinal issues and pulmonary arterial hypertension (PAH).

#### 2.1.2 Diffuse cutaneous systemic sclerosis

a cutaneous sclerosis involving the face, proximal parts of the limbs and trunk. May be present with tendon friction rubs. Associated with topoisomerase I and RNA polymerase III. The course of this type of scleroderma is more severe - rapid progression of skin fibrosis, early onset of renal, pulmonary and cardiac complications;.

#### 2.1.3 Systemic sclerosis sine scleroderma

involvement of internal organs with sparing of the skin. Mostly occurrence of Raynaud’s phenomenon, PAH and nailfold capillary abnormalities (1, 2)..

#### 2.1.4 Overlap syndromes

disorders wherein the classification criteria of at least two distinct connective tissue diseases are met (1).

The etiology still remains unknown. Genetic factors (including HLA DRB1*1104, DQA1*0301 and DQB1*0501, or PTPN22, IRF5, STAT4 or NLRP1) and environmental factors (silica dust, parvovirus B19 infection, CMV, EBV, certain drugs) are suspected to play a significant role in the development of the disease.

The pathogenesis of the disease is also not fully understood. One mechanism for the development of the disease is damage to the vascular endothelium and the release of ET-1, which impairs vascular haemostasis, leading to platelet activation, thrombus formation, coagulation abnormalities, increased leukocyte adhesion, vasoconstriction resistant to vasodilator compounds such as nitric oxide or prostacyclins and vasoconstriction. Ultimately, microangiopathy, vascular remodeling and loss, and impaired blood flow and tissue hypoxia occur.

Another mechanism considered is the activation of T lymphocytes with a predominance of Th2 lymphocytes, which increase the production of pro-inflammatory and pro-fibrotic cytokines, including interferon-γ, TGF-β, IL-4, IL-5, IL-13. Blood-circulating macrophages, dendritic cells and monocytes are also involved in the development of the disease. As a result of the mechanisms described above, tissue fibrosis and the following symptoms occur.

In 95% of patients, Raynaud’s phenomenon is found. The skin of the fingers becomes ulcerated as a result of impaired vascularisation, atrophy of the fingertips and, in more severe cases, ischaemia of the fingers and gangrene formation. The nature of the skin involvement changes over time, allowing 3 phases to be distinguished: oedema, induration and atrophy.

Initially, due to inflammatory swelling, the fingers have a ‘sausage-like’ appearance (puffy fingers). Patients complain of pain, itching and erythema. In addition, nerve compression may occur, resulting in the manifestation of neuropathy symptoms including carpal tunnel syndrome.

This is followed by skin fibrosis, thickening and hardening (sclerodactyly). The skin is tight and contractures occur in the joints with their limited mobility.

The involvement of facial skin causes a mask-like face with narrow lips and an inability to open them wide. As a result of the disruption of melatonin distribution, there is hyperpigmentation with areas of hypopigmentation - salt- and- pepper-like appearance. Other skin manifestations include telangiectasias and calcifications in areas prone to trauma, mainly the extensor parts of the elbow joints and fingertips.

### 2.2 In addition, the course of the disease includes

· Joint and muscle pain, muscle weakness, reduced joint mobility, arthritis - mainly in the hands, wrists and ankles. In 30% of cases, mainly dSSc, tendon fibrosis with tendon damage is observed. Muscle fibrosis and muscle atrophy may also occur. Myopathy and tendon involvement are harbingers of a poor prognosis.
· Gingivitis, periodontitis, dry mouth (as a symptom of secondary Sjogren’s syndrome due to salivary gland fibrosis), dysphagia, symptoms of gastroesophageal reflux disease. Gastroparesis and its consequences - bloating, early satiety, nausea, anorexia, malnutrition and weight loss. In addition, watermelon stomach and increased gastrointestinal bleeding may occur. Disturbed peristalsis leads to diarrhea, constipation, the development of SIBO syndrome and even pseudo-obstruction. Coexistence of systemic sclerosiswith primary biliary cirrhosis of the liver is observed in some individuals.
· Interstitial lung disease, pulmonary hypertension, less commonly pleuritis, airway hemorrhage, cryptogenic organizing pneumonia, aspiration pneumonia.
· Cardiac arrhythmias (usually ventricular premature beats, but also supraventricular tachycardia, atrioventricular block, intraventricular conduction disturbances), pericarditis, rarely tamponade; dilated cardiomyopathy, left ventricular diastolic dysfunction due to pulmonary hypertension.
· Sclerosing renal crisis (SRC) - observed in approximately 10% of patients within 3 years of diagnosis. It is characterized by rapidly increasing hypertension or the onset of malignant hypertension, microangiopathic haemolytic anemia, thrombocytopenia, moderate proteinuria and erythrocyturia. Risk factors for renal scleroderma include dSSc, use of high-dose corticosteroids or chronic steroid therapy, newly diagnosed anemia, pericardial effusion and the presence of antibodies to RNA polymerase III.
· Hypothyroidism
· Depression

Patients with scleroderma are at increased risk of developing other autoimmune diseases (including secondary Sjögren’s syndrome, primary biliary cirrhosis of the liver), as well as Graves Basedow’s disease and Hashimoto’s inflammation co-morbidities (3).

The diagnosis is established based on the ACR/EULAR 2013 classification criteria for systemic sclerosis as shown in **Table 2** (4).

**Table 2:** The ACR / EULAR Criteria for the classification of Systemic Sclerosis (4).

Patients having a total score ≥9 points are being classified as having definite systemic sclerosis.
• Skin thickening of the fingers of both hands extending proximal to the metacarpophalangeal joints: 9 points • Skin thickening of the fingers*: • Distal to MCP but proximal to the PIPs - Sclerodactyly of the fingers: 4 points • Puffy fingers: 2 points • Finger tip lesions*: • Finger Tip Pitting Scars: 3 points • Digital Tip Ulcers: 2 points • Teleangiectasia: 2 points • Abnormal nailfold capillaries: 2 points • Raynaud’s phenomenon: 3 points • arterial hypertension and/or Interstitial lung Disease: 2 points • Scleroderma related antinuclear antibodies: Anti-centromere, Anti-topoisomerasel, Anti-RNA polymerase III: 3 points
* only count the highest score

The diagnosis of the disease is complex. An assessment of the severity of skin involvement should be performed using the modified Rodnan scale (mRSS), where 0 is no change and 3 is severe skin thickening. Assessment of the nail shafts by capillaroscopy is also recommended.

In laboratory tests, antinuclear antibodies are found in 90% of patients, and typical antibodies for systemic sclerosis (anti-Scl70, anti-centromere, anti-RNA polymerase III) are found in about 70% of patients. Anti-centromere antibodies are mainly present in a restricted form. Their presence is associated with a higher risk of pulmonary arterial hypertension, but a reduced risk of interstitial lung disease and limited skin involvement. Antibodies to topoisomerase I (Scl 70) are more common in the generalized form. Their detection is associated with a higher risk of interstitial lung disease, symptoms indicative of cardiac involvement and diffuse skin involvement. Antibodies to RNA polymerase III are more frequently detected in the generalized form. Patients with these antibodies have rapid disease progression and severe skin involvement with less frequent respiratory involvement. In addition, they may have an increased risk of malignancy. Other antibodies that may be present in systemic sclerosis include anti-U3-RNP, anti-Th/To and anti-PM/Scl antibodies.

As a follow-up diagnosis, radiographs of the hands should be taken to show osteolysis of the distal phalanges and calcinosis. If pulmonary involvement is suspected, respiratory function tests and a chest CT scan are recommended. Investigations to assess cardiovascular involvement include echocardiography, electrocardiography, Holter ECG and cardiac MRI. In case of gastrointestinal symptoms, i.e. dysphagia, upper gastrointestinal endoscopy should be performed, and esophageal manometry, gastrointestinal contrast study with barium, 24-hour ph-metry may also be performed.

Early recognition and initiation of treatment allows for clinical improvement. For each patient, education, setting treatment goals, adherence to a healthy diet, regular physical activity and psychological assessment and support should be sought. Pharmacological therapy is based on the symptoms and organ involvement. Management should be dependent on the organ involvement in question.

If Raynaud’s phenomenon is found, patients should avoid exposure to cold, stress, nicotine and the use of sympathetic nervous system stimulants or beta-blockers. The mainstay of pharmacological therapy is the use of vasodilators such as calcium channel blockers (amlodipine, nifedipine), pentoxifylline, phosphodiesterase type 5 inhibitors, nitroglycerine, prostacyclin analogues (iloprost) or endothelin receptor antagonists (bosentan). The main aim of treatment is to prevent ulceration and necrosis of the fingers.

Immunosuppressive drugs are used to treat skin lesions, including methotrexate, mycophenolate mofetil, cyclophosphamide and hydroxychloroquine.

For musculoskeletal involvement, analgesic therapy with non-steroidal anti-inflammatory drugs, methotrexate, hydroxychloroquine is recommended. Myositis is treated with azathioprine or methotrexate.

In the treatment of interstitial lung disease, improvements are observed after cyclophosphamide, mycophenolate mofetil and nintedanib.

The 2023 updates of EULAR recommendations for the treatment of systemic sclerosis (SSc) encompass a comprehensive review of new evidence and therapeutic questions. A diverse task force of 27 members, including patients and methodologists, prioritized 67 clinical questions across 24 interventions. These updates, spanning 8 clinical domains, introduce 21 recommendations, notably including the use of mycophenolate mofetil, nintedanib, rituximab, and tocilizumab for key manifestations such as skin fibrosis and interstitial lung disease. These advancements reflect significant progress in addressing crucial aspects of SSc management and provide clinicians with stronger evidence to guide treatment decisions (5).

Cardiac arrhythmias are mainly treated with antiarrhythmic drugs and, when indicated, with the implantation of a cardioverter defibrillator.

The use of proton pump inhibitors and prokinetic drugs reduces gastrointestinal complaints. Antibiotic therapy is used in patients with small bowel bacterial proliferative syndrome.

In cases of scleroderma renal crisis, the only effective treatment is the administration of angiotensin-converting enzyme inhibitors (ACEIs). Captopril is the preferred drug due to its short duration of action and flexibility in dosing. When a patient manifests symptoms of increasing renal failure, dialysis therapy should be initiated. The use of prophylactic ACEIs is not recommended due to their lack of efficacy and adverse impact on prognosis.

Due to the increased risk of scleroderma renal breakthrough during treatment with corticosteroids, it is recommended to use them only when necessary.

At present, there is no causal treatment for the disease. Due to the presence of symptoms from multiple systems, a multidisciplinary approach and appropriate education of the patient is necessary (3).

## 3 Morphea

Morphea, or localized scleroderma, is an inflammatory disease of the skin and subcutaneous tissue. The incidence is estimated to be between 3.4 and 27 per 100 000. It affects both children and adults, more often women and Caucasians. The peak incidence in adults is seen in 40-50 years of age and in children 7-11 years of age. The etiology of the condition is unknown. Several factors are suspected to play a role in its onset, including environmental, genetic and immunological factors. Genetic factors include the HLA DRB1*04:04 and HLA-B*37 genes found in the patients. Serum ANA, anti-histone antibodies and anti-SSDNA antibodies, as well as a positive family history of autoimmune diseases, point to an autoimmune background. In addition, external trauma or exposure to ionizing radiation has been attributed a role in the pathogenesis of the disease.

Under the influence of the aforementioned factors, excess collagen is deposited in the dermis and blood vessel function is impaired (6). Damage to the vascular endothelium leads to increased production of IL-4, as well as Il-6, TGF- β, activation of macrophages and T lymphocytes by intracellular cell adhesion molecule 1 (ICAM1) and vascular cell adhesion molecule 1 (VCAM1). The above mechanisms lead to fibrosis and worsening of vascular damage (6, 7).

The clinical manifestations mainly concern the skin. Initially, the lesions are erythematous and accompanied by tenderness and pruritus. With time, they become hard and surrounded by a purple border. Eventually, atrophic lesions with excessive or reduced pigmentation develop. The lesions may regress spontaneously within a few years, although this is not the case in all cases (6).

### 3.1 Due to the varied clinical picture, localized scleroderma can be divided as follows:

#### 3.1.1 Linear scleroderma (linear morphea)

band-like lesions, located on the head, neck and extremities. Onset usually in childhood - most common variant in children. In this form, deeper layers may be involved, leading to contractures in the joints, underdevelopment of the limbs in children or muscle weakness.

#### 3.1.2 Morphea of the ‘en coup de sabre’ type (the blow of a sword)

the lesion involves the hairy scalp, giving it the characteristic appearance of a sabre cut. Half facial atrophy can be observed in this form. Some patients manifest neurological symptoms such as convulsions and headaches.

#### 3.1.3 Circled morphea

an isolated focus of sclerosis, most commonly occurring on the chest and trunk. There are two types: superficial - limited to the dermis; and deep - fibrosis and scarring of the subcutaneous tissue, fascia and muscles, which can result in restricted mobility and contractures in the joints.

#### 3.1.4 Generalized morphea

confluent plaque-like induration of the skin, a minimum of 4 foci of induration in two or more different locations. Its course is dangerous and can lead to contractures in the joints.

#### 3.1.5 Other rare forms

papular scleroderma (morphea guttate) and bullous scleroderma (morphea bullous)

#### 3.1.6 Mixed scleroderma (mixed morphea)

coexistence of 2 or more types (6). It occurs in 15% of patients (7).

The diagnosis is usually made on the basis of the clinical picture. Laboratory tests may show peripheral eosinophilia and elevated inflammatory parameters. In patients with a severe course, ultrasound and MRI may be performed to determine the involved area (6). Occasionally, a skin biopsy including adipose tissue and, if required, deeper layers is performed to confirm the diagnosis and differentiate from scleroderma-like syndromes. Histopathological examination reveals an inflammatory infiltrate composed of lymphocytes, eosinophilia, macrophages and plasma cells; advanced lesions show collagen deposits in the reticular layer, blood vessels and eccrine glands, as well as atrophy of fat cells and their replacement by areas of fibrosis (6, 8).

In the case of co-occurrence of Raynaud’s phenomenon and the presence of antinuclear antibodies, the diagnosis of systemic sclerosis should be deepened.

The essence of effective treatment is to start therapy at an early stage of the disease, this allows the occurrence of complications of the disease to be limited. Superficial lesions are treated with topical glucocorticosteroids or, less commonly, tacrolimus 0.1% for 3-4 weeks with good results. First-line therapy for extensive lesions is UVA1 phototherapy, possibly PUVA, broadband UVA, narrowband UVB. For aggressive lesions, systemic corticosteroids (oral or intravenous) or methotrexate are recommended. In case of symptom recurrence after completion of steroid therapy, combination therapy of glucocorticosteroids with methotrexate is recommended. When patients cannot tolerate methotrexate preparations, they can be replaced by mycophenolate mofetil. In some cases - mainly morphea of the ‘en coup de sabre’ type - surgical treatment is undertaken.

In the differential diagnosis, attention should be paid to whether the patient has symptoms typical of systemic sclerosis - Raynaud’s phenomenon, antinuclear antibodies, telangiectasias, calcinosis, capillaroscopic changes, involvement of internal organs - e.g. lungs, heart, esophagus. As previously mentioned, histopathological examination of a skin specimen is helpful in the differential diagnosis with scleroderma-like syndromes.

In most cases, the prognosis is good (6). Morphea whose onset is in the early years of life lasts longer, recurs more frequently or predisposes to a chronic course (7). The aggressive progression of the disease and involvement of deeper layers can lead to contractures in the joints and limit joint mobility (6).

## 4 Inflammatory diseases

### 4.1 Eosinophilic fasciitis Schulman

Eosinophilic fasciitis Schulman is an autoimmune disease characterized by painful swelling and induration of the skin usually involving the upper and lower limbs. In some cases, the inflammatory process can also involve internal organs such as the lungs, heart, kidneys or gastrointestinal tract (9). It occurs mainly in people between 20 and 60 years of age, more often in men (1.5:1) (10). The etiology is still unknown. Cases have been reported in the literature of people who developed symptoms after exercise, trauma, childbirth, radiotherapy, Borrelia burgdorferi infection, insect bites and the use of certain medications - including statins (8, 10, 11).

An autoimmune process is suspected as the cause of the disease development. This is supported by stimulation of the immune system (hypergammaglobulinaemia), a good response to steroid therapy and the presence of rheumatoid factor, antinuclear antibodies and immune complexes in the patient’s serum.

The role of overexpression of type I collagen and fibronectin by dermal fibroblasts is considered in the pathophysiology (10). Due to elevated levels of eosinophilia, their increased migration, eosinophilic cationic proteins and IL-5, it is suspected that eosinophils are responsible for the initiation of the inflammatory process (8, 10). Furthermore, increased production of tissue inhibitor of metalloproteinase-1 (TIMP-1) has been found to stimulate fascial fibrosis. Activation of mast cells with increased histamine, IL-2, interferon-γ (IFN-γ) and leukemia inhibitory factor (LIF), excessive CD40 activation and increased superoxide dismutase (SOD) levels were also noted. Laboratory studies detected an elevated fraction of Th17 cells, TGF-β1 and connective tissue growth factor gene expression (10).

The onset of symptoms is often preceded by heavy exercise or childbirth. In most patients, the development of the disease is slow - over weeks to months. Patients complain of symmetrically occurring swelling with induration (woody induration) of the distal parts of the extremities with sparing of the hands and face (12). Symptoms may be accompanied by redness, pain, fever, generalized fatigue and weight loss. Patients’ skin takes the form of an orange peel (‘peau d’orange’) and there is a characteristic groove sign at the course of the superficial venous vessels. Inflammatory involvement of the fascia leads to reduced joint mobility and results in an inability to fully extend the fingers at the joints (prayer sign) (7). Due to pressure on the median nerve, patients report symptoms of carpal tunnel syndrome - tingling, numbness in the area of the first, second, third and half of the fourth finger of the hand (10). On physical examination, induration of the subcutaneous tissue without involvement of the upper layers of the skin is felt, causing the examiner to be able to grasp the skin with two fingers causing it to crease. Occasionally, patients may present with Raynaud’s phenomenon (11). Arthritis of the hands and knees may also occur during the course of the disease (12).

In 2017, M. Jinnin et al. distinguished classification criteria for eosinophilic fasciitis - see **Table 3**. In addition, they developed criteria to assess the severity of the disease - see **Table 4** (13).

**Table 3:** Diagnostic criteria of eosinophilic fasciitis (13).

Major criterion
Symmetrical plate-like sclerotic lesions are present on the four limbs. However, this condition lacks Raynaud's phenomenon, and systemic sclerosis can be excluded
Minor Criteria 1
The histology of a skin biopsy that incorporates the fascia shows fibrosis of the subcutaneousconnective tissue, with thickening of the fascia and cellular infiltration of eosinophils and monocytes.
Minor Criteria 2
Thickening of the fascia is seen using imaging tests such as magnetic resonance imaging (MRI).
A DEFINITVE DIAGNOSIS: MAJOR CRITERION + AT LEAST 1 OF THE MINOR CRITERIA

**Table 4:** Severity classification of eosinophilic fasciitis (13).

Joint contracture of upper limb	1 point
Joint contracture of lower limb	1 point
Limited movement in upper limbs	1 point
Limited movement in lower limbs	1 point
Expansion and worsening of skin rash – progression	1 point
≥2 points – severe eosinophilic fasciitis	

Pinal- Fernandez and colleagues also proposed establishing a diagnosis based on the fulfillment of the criteria listed below:

· Greater criteria: oedema, thickening and induration of the skin and thickening of the subcutaneous tissue and fascia observed in the skin and fascia biopsy with infiltration of lymphocytes and macrophages;
· minor criteria: hypergammaglobulinaemia, peripheral eosinophilia, elevated aldolase and/or muscle weakness, peau d’orange sign and/or groove sign, hyperintense fasciculations on T2- dependent MR images.

The diagnosis is confirmed by meeting all major criteria or by meeting one major and two minor criteria (12).

Histopathological examination of a section of fascia with skin plays a key role in the diagnosis. Macroscopically, the fascia is thickened, lymphocytes and plasma cells are observed microscopically, and eosinophils are found in 50% of patients at an early stage. The damage is mainly to the fascia, but in more severe cases swelling and proliferation of collagen fibers located in the skin may occur (10).

Laboratory blood tests are dominated by eosinophilia, accelerated ESR, hypergammaglobulinaemia, and elevated aldolase levels, which correlate with disease severity (8, 10). Eosinophilia is not necessary for diagnosis as it is only present in a proportion of patients, mainly at the beginning of the disease. In addition, serum type III procollagen peptide (PIIIP) is present. In about 10% of patients, antinuclear antibodies and rheumatoid factor are detected in significant titres (10). MRI and PET-CT are helpful tools for imaging the involved fascicles and assessing treatment efficacy (9).

In the differentiation from systemic sclerosis, the clinical picture is particularly important. Eosinophilic fasciitis mainly involves the fascia, whereas systemic sclerosis involves the skin, although fascia, muscle and bone may also be involved (10). In eosinophilic fasciitis, in contrast to scleroderma, there is no characteristic capillaroscopy picture, no thickening of the distal phalanx area, no ulceration of the fingers and no involvement of internal organs (14).

It is also worth mentioning eosinophilic fasciitis in the context of paraneoplastic syndrome. Cases of fasciitis in the course of hematological (10-15% of cases)], breast, lung, prostate and melanoma cancers have been reported in the literature (12, 15).

The disease may go into spontaneous remission (12). First-line treatment is oral steroid therapy, starting at 20-30 mg/d and reducing the dose according to clinical symptoms, i.e. skin hardness, joint mobility (10). Typically, treatment lasts 12-18 months (12). The efficacy of the therapy is estimated at 90% and the prognosis is good (10). However, some patients experience a relapse during steroid dose reduction (9). In such cases, it is recommended to start second-line treatment - cyclosporine, cyclophosphamide, methotrexate (10). Individual studies have shown the efficacy of combining steroid therapy with mycophenolate mofetil, azathioprine, sirolimus, JAK-kinase inhibitors, dapsone, tocilizumab, infliximab, rituximab, as well as the administration of intravenous immunoglobulin and antithymocyte globulin (14–16). In addition, psoralen and ultraviolet A (PUVA) have been used in therapy (10).

In most cases, the prognosis is good. Late initiation of immunosuppressive treatment is associated with complications, i.e. irreversible contracture in the joints, so treatment should be started as soon as possible if a diagnosis is made (9).

### 4.2 Lichen sclerosus

Lichen sclerosus is a chronic inflammatory dermatosis, more common in women (F:M 5:1). 50% of described cases are postmenopausal women, 40% women of reproductive age and 9% pre-pubertal age. In the female sex, two periods of onset of the first symptoms are distinguished - prepubertal girls and postmenopausal women. The onset of the disease in the male sex is 40-50 years. Risk factors for the disease include: Caucasian race, positive family history, presence of HLA DQ7, HLA DQ8, HLA DQ9, HLA 11 and HLA 12 genes, burden of other autoimmune diseases such as autoimmune thyroiditis, vitiligo, alopecia areata, or pernicious anemia, presence of a foreskin, urinary incontinence, condition after trauma or natural childbirth, local treatments, use of oral contraceptive pills with anti-androgenic effect, presence of Koebner’s sign. In addition, cases of lichen sclerosus in patients treated with carbamazepine and imatinib and its co-occurrence with morphea have been reported. Infection with HPV, HCV, EBV is also suspected to be a cause of the disease (17).

The pathogenesis of the disease is not fully understood, the main cause is thought to lie in the activation of fibroblasts by interleukin 4 and TGF β, which successively stimulate collagen production and lead to tissue fibrosis. Also, it has been hypothesized that a decrease in CD3+ T lymphocytes and an increase in macrophages, mast cells, CD4+ monoclonal T lymphocytes, CD1a+ dendritic T lymphocytes, interleukin 1 and interleukin 1 antagonist receptors may influence the development of the disease (18). The discovery of anti-BP180 and anti-BP230 antibodies and IgG class antibodies to extracellular matrix protein 1 in the serum of people with impetigo may also play a significant role in the complex process of skin lesion formation (17).

The disease mainly affects the anogenital region - in women the perineum, vulva and anus, in men the glans and foreskin area, but lesions on other areas of the body are also observed in 15-20% of patients. Oral mucosal involvement is relatively rare - oral lichen sclerosus (18). Initially, the lesion is well-demarcated, erythematous and oedematous, successively becoming a pale, thin and hard area. The atrophic skin is prone to injury and rupture, resulting in multiple erosions and petechiae and eventually leading to scarring (17). When the vulva, perineum and perianal are involved, a characteristic keyhole sign is noted (17, 19).

The specific lesion is lichen-like porcelain-white or ivory-coloured papules and atrophic plaques with hyperkeratosis and hypopigmentation. In men, there may be a history of sciatica and, if the urethra is involved, fibrosis, stricture and urinary disturbances or even retention. Patients complain of itching, burning, pain, dysuria, painful defecation and dyspareunia. Some patients may not present with symptoms; however, this does not equate to a benign course of the disease (17).

Lesions outside the anogenital area are mainly found on the neck, shoulders, upper back, under the breasts, wrist area and thighs (19).

In order to confirm the diagnosis established by the clinical picture, a skin biopsy is performed. The changes observed on histopathological examination are: lymphocytic infiltration in the basal layer of the epidermis, basal cell hydrotic degeneration, subepithelial oedema and homogenisation of the connective subepidermal layer, hypopigmentation with collagen homogenisation, atrophy and superficial hyperkeratosis. Blood vessels are dilated and may present features of lymphocytic or lymphohistiocytic vasculitis. On histopathological examination, a proliferative process should be excluded, as 4-7% of individuals develop transformation of the disease into squamous cell carcinoma.

The first line of treatment is topical glucocorticosteroid preparations - 0.05% clobetasol propionate ointment applied for 3 months. A good response to this treatment is usually achieved. Other drugs used are the calcineurin inhibitors - pimecrolimus and tacrolimus. These have been shown to be less effective compared to clobetasol propionate. Therapy also includes topical or oral retinoids, combination therapy of methotrexate (10-15 mg/week) with GCS, cyclosporine (2-4 mg/kg/day), TNF-alpha inhibitors i.e. adalimumab (single injection into the affected area). PUVA phototherapy is also recommended. In the absence of a response to these therapies and the occurrence of complications in the form of a stool or urethral stricture, surgery is the treatment of choice. It is worth remembering that surgery can only be performed in cases of inactive disease (17).

Lichen planus, lichen simplex chronicus, eczema, morphea, psoriasis, child sexual abuse, contact dermatitis and vitiligo should be considered in the differential diagnosis.

Promptly making a correct diagnosis and initiating treatment increases the chance of a benign course of the disease, without developing complications (18).

### 4.3 POEMS syndrome

Features of POEMS syndrome (osteosclerotic myeloma) include polyneuropathy, organomegaly (enlargement of the liver, spleen or lymph nodes), endocrinopathies, monoclonal gammopathy (almost always light λ chains) and skin lesions. The above symptoms may be accompanied by osteosclerotic fractures, oedema, pleural fluid, thrombocytosis, polycythaemia and Castelman’s disease. In 90% of patients, skin lesions are present, including thickening of the skin - mainly of the hands and feet (19, 20); sclerodactyly (16% of cases) (12), hyperpigmentation, telangiectasias, excessive hairiness and glomus haemangiomas (19, 20). Raynaud’s phenomenon is observed in 20% of patients (12). The skin lesions are accompanied by abnormalities in the examination of respiratory mechanics. The prevalence of the disease in the population is estimated to be 0.003%, more commonly in men (19, 20) aged 60-70 years (12).

Pro-inflammatory cytokines - VEGF, IL-1, IL-6 - play a major role in the pathogenesis of POEMS syndrome and VEGF is suspected to be responsible for skin thickening. Determination of plasma or serum VEGF levels is one of the diagnostic criteria and is used to monitor the effectiveness of treatment.

If the diagnosis is made quickly and treatment is initiated, the prognosis is good. The diagnosis is made based on the diagnostic criteria - see **Table 5**.

**Table 5:** Diagnostic criteria for poems syndrome (21).

Mandatory criteria
• Polyneuropathy (typically demyelinating)
• Monoclonal plasma cell-proliferative disorder (almost always λ)
Major criteria
• Castleman disease
• Sclerotic bone lesions
• Vascular endothelial growth factor (VEGF) elevation
Minor criteria
• Organomegaly (splenomegaly, hepatomegaly, or lymphadenopathy)
• Extravascular volume overload (edema, pleural effusion, or ascites)
• Endocrinopathy (adrenal, pituitary, gonadal, parathyroid, thyroid and pancreatic)
• Skin changes (hyperpigmentation, hypertrichosis, glomeruloid hemangiomata, plethora, acrocyanosis, flushing, and white nails)
• Papilledema
• Thrombocytosis/polycythemia
Diagnosis of POEMS syndrome: The two mandatory criteria + ≥ 1 major AND ≥ 1 minor criterion

In the differential diagnosis with scleroderma, the presence of serum monoclonal protein, organomegaly, peripheral neuropathy, excessive hairiness and glomerular hemangiomas are helpful. In addition, the absence of antinuclear antibodies and capillaroscopy changes supports the diagnosis of POEMS syndrome.

The primary treatment of the disease is autologous haematopoietic cell transplantation in patients eligible for this procedure, while others are given dexamethasone with melphalan or with lenalidomide/thalidomide (19, 20).

### 4.4 GvHD

Patients who undergo allogeneic bone marrow, stem cell or, less commonly, solid organ transplantation may develop graft-versus-host disease within a year (19). They usually present with a range of symptoms due to hepatitis, intestinal and skin inflammation, although there are cases of patients whose only manifestation of the disease is the development of scleroderma-like lesions (7).

To date, the pathogenesis of the condition is not fully understood. T-cell activation appears to be the main cause of the disease. Under the influence of CD4 and CD8 lymphocytes, INF γ is released, which stimulates fibroblasts to overproduce collagen. It has also been observed that patients who are older, have chronic myeloid leukemia, have a higher HLA mismatch with their donor, have HLA - A1, HLA - B1, HLA - B genes, have had a splenectomy, CMV infection, hemiplegia infection, or who are male and their donor was female have a higher risk of GvHD and skin involvement (7).

The clinical picture is complex. One of the symptoms may be localized or generalized progressive thickening and hardening of the skin, which is dry and itchy. In the course of the disease, fascial involvement and the appearance of a peau d’orange skin crease, groove sign and restricted mobility and contractures in the joints - the ‘prayer sign’ - may occur (19). In addition, skin ulcerations may occur during the course of the disease. Other skin lesions include localized scleroderma-like lesions, lichen sclerosus and lichen planus. Localized skin involvement is most commonly seen on the trunk and proximal parts of the extremities. In lesions similar to morphea and lichen planus, the predominant area is the lower torso. These lesions may merge together to form large areas of sclerosis (12).

Differentiation from scleroderma can be challenging. The capillaroscopic picture may resemble scleroderma microangiopathy. Disease also like in scleroderma may lead to the development of calcinosis (19). Antinuclear antibodies are usually not found in laboratory tests, although in some cases anti-PM-Scl, anti-Scl-70, anti-La, ANCA or APLA antibodies are present in the serum of patients (7, 8, 19). Histopathological examination presents with thickened epidermis, thick collagen fibers that may infiltrate subcutaneous tissue and fascia, perivascular lymphocyte infiltration, loss of perivascular fat cells and hyperkeratosis. Sometimes the picture can be identical to that of scleroderma, making differentiation much more difficult (8, 19).

As the condition is a complication of transplantation, a meticulously taken history plays a major role in diagnosis. Patients deny the presence of Raynaud’s phenomenon, and the finding of hepatitis, intestinal inflammation, mucosal lesions, scalp, hair, genitalia or dry eyes argue against the diagnosis of systemic and limited sclerosis (19).

First-line treatment is a modification of immunosuppressive treatment - mainly a combination of prednisone and tacrolimus. Other therapies with proven efficacy are extracorporeal photopheresis, daclizumab, pentazocine, mycophenolate mofetil, sirolimus, rituximab, imatinib (12).

## 5 Deposition disorders

### 5.1 Nephrogenic systemic fibrosis

Nephrogenic systemic fibrosis is a progressive life-threatening disease affecting people with renal failure, most commonly after exposure to gadolinium contrast agents (GdCA).

The onset of disease depends on the degree of renal damage and the type and dose of contrast agent used. Significant risk factors for the development of symptoms include G5-G3 stage of chronic kidney disease, contrast dose >0.3mmol/kg, total contrast concentration given during life and a linear, non-ionic form of contrast agent. It is also suspected that high phosphate concentrations, ongoing inflammatory process in the body and use of beta-blockers may increase the likelihood of disease (22). Exposure to gadolinium contrast agents is a key factor leading to the development of nephrogenic systemic fibrosis, although cases of disease have been recorded in patients who never received the above agents (23). Currently used linear and macrocyclic compounds with greater molecular stability (gadoxetate disodium and gadobenate dimeglumine) have resulted in a significant reduction in cases of nephrogenic systemic fibrosis (12).

Excretion of gadolinium contrast agents is mainly via the kidneys. In individuals with abnormal renal function, the time taken to remove contrast from the body is prolonged compared to healthy individuals, resulting in the breakdown of gadolinium chelate, which is a relatively safe form, and the formation of the toxic form, free gadolinium cation. This compound, by activating monocytes and macrophages, leads to the release of chemokines and cytokines that stimulate fibroblasts and activate the fibrosis process. In addition, it stimulates the maturation of dendritic cells and the production of TGF- β1, resulting in the deposition of collagen in tissues and increased fibrosis (22). Based on a study by Mackay - Wiggan et al. anticardiolipin and antiphospholipid antibodies were found to also be involved in the development of nephrogenic systemic fibrosis. In addition, in response to oedema and immunosuppression, patients with antiphospholipid antibodies experience fibroblast activation and deposition of mucin deposits in the skin, resulting in skin hardening and thickening (24).

The onset of symptoms is usually observed within 2-10 weeks of contrast agent administration. Initially, patients report burning pain, weakness, swelling, redness and pruritus with associated papules or plaques around the distal parts of the extremities. Subsequently, the lesions spread to proximal areas with sparing of the facial skin and resolution of the swelling. Subsequently, the skin becomes hard, thickened and shiny and areas of hyperpigmentation appear. Its appearance is compared to that of an orange peel. Patients complain of a feeling of stiff skin on the hands (tight glove symptom) and reduced joint mobility (22). In the final stage, skin atrophy and hair loss occur (23). In some cases, deposition of calcium deposits in the tissues and the occurrence of cutaneous calcinosis have been observed (22).

In addition, fibrosis of internal organs, i.e. lungs, glomeruli, liver, esophagus, heart, skeletal muscle or dura mater, was found in less than 5% of patients (19).

The diagnosis is established on the basis of the clinical picture, the differential diagnosis and the histopathological result of the full-thickness skin section. Histopathology reveals features of fibrosis involving the entire skin with a clear demarcation from adipose tissue, without features of inflammatory infiltration. In addition, thickened and thin collagen fibers, dermal spindle shaped, mucin deposits and CD34 stem cells are seen. In some patients, when a dermatomyosarcoma biopsy is performed, muscle cell fibrosis involvement can also be observed (19, 22).

When differentiating from systemic sclerosis, it is important to note that in nephrogenic systemic fibrosis, patients usually do not present Raynaud’s phenomenon, sclerodactyly, features of arthritis and antinuclear antibodies (19).

Treatment of nephrogenic systemic fibrosis remains a challenge, which is why prevention of disease onset is very important. The use of intravenous immunoglobulin, a combination of methotrexate and glucocorticosteroids with PUVA phototherapy or topical interferon relieves skin symptoms. Another therapy leading to clinical improvement is extracorporeal photopheresis. However, kidney transplantation is so far the only effective and proven treatment option. In patients after transplantation, inhibition of disease progression and regression of lesions have been observed (22, 23).

### 5.2 Scleromyxedema

Scleromyxedema is a primary cutaneous mucinosis. It is one of the chronic rare diseases with an equal incidence in both sexes and a mean age of first symptom manifestation of 59 years. The etiology so far remains unknown (25). It can coexist with systemic connective tissue diseases, including systemic sclerosis, HCV infection, HIV infection and hematological malignancies (12). The diagnosis is based on meeting 3 of 4 features: presence of a generalized papular, sclerodermic rash, mucin deposits in the skin, fibroblast proliferation and increased collagen found on histopathological examination of a skin specimen, co-occurrence of monoclonal gammopathy - mainly IgG lambda, exclusion of thyroid dysfunction (25).

The pathogenesis of the disease is not fully understood; it is suspected that TGF-β, TNF-α, IL-1 circulating in the blood, which activate fibroblasts to produce mucin, may be responsible for the fibrosis. Furthermore, paraproteinaemia detected in laboratory tests may also be involved in the formation of skin lesions (12).

The clinical picture is dominated by swelling, erythema and small, dome-shaped, white and waxy papules arranged in a linear pattern. The skin is furthermore thickened, hardened, with a ‘cobblestone’ texture. This leads to microstomia, sclerodactyly and contractures in the joints (19, 25). The lesions are mainly located on the face, retroauricular region, neck, dorsal part of the hands and upright part of the forearms with sparing of the scalp, palms and mucous membranes (12, 19, 25). The nodules fuse together to give the appearance of a lion’s face and the occurrence of the Shar-Pei sign on the trunk (19, 25). In addition, the thickening of the facial skin results in reduced elasticity and reduced mouth opening (8). In the area of the proximal interphalangeal joints, thickening of the skin develops, with central concavity causing the ‘doughnut’ sign (19, 25).

70% of patients develop extracardiac manifestations - dysphagia, dyspnoea, pulmonary hypertension, features of restriction or obstruction observed on respiratory mechanics testing, myocardial ischaemia, pericardial fluid, scleroderma-like renal damage, muscle weakness, inflammatory myopathy, fibromyalgia, peripheral arthritis, epileptic seizures, peripheral neuropathy, aphasia, carpal tunnel syndrome, depression, psychosis and dementia. In rare cases, scleromyxedema can lead to the development of ‘dermato-neuro syndrome’. This is a syndrome of neurological symptoms, the onset of which is preceded by flu-like symptoms. The clinical picture includes encephalopathy that can lead to coma and even death.

Oncological vigilance should be exercised during diagnosis, as transformation of scleromyxedema into multiple myeloma has been observed in 10% of patients (19, 25).

The modified Rodnan scale for scleromyxedema (mRSSS) is used to assess disease severity. It involves examining 20 different skin areas for the extent of papules, the degree of skin thickening, the severity of erythema and the presence of paresthesia or pruritus (25).

One of the diagnostic criteria mentioned earlier is the histopathological examination of the skin biopsy. In the case of scleromyxedema, collagen fibers divided by amorphous material and an inflammatory infiltrate composed of plasma cells and lymphocytes are found (11). In addition, the image shows mucin and hyaluronic acid deposits (8). Mucin deposits can also be detected in biopsy specimens of involved internal organs, i.e. kidneys, lymph nodes or heart (12).

Furthermore, for the differential diagnosis of skin lesions with systemic sclerosis, a meticulous physical examination and complementary laboratory tests should be performed. Compared to systemic sclerosis, patients do not show telangiectasia, Raynaud’s phenomenon, features of calcinosis, characteristic microangiopathy on capillaroscopy or the presence of antinuclear antibodies (25). Signs supporting the diagnosis of scleromyxedema include the presence of papules, ear involvement and mid-back involvement (11).

The preferred treatment is high-dose intravenous immunoglobulin (2g/kg over 5 days every 4-6 weeks). If there are contraindications or if treatment is not effective, thalidomide (400mg/d) or lenalidomide (25mg/d for 3 weeks per month) is recommended as monotherapy or in combination with steroid therapy. Bortezomib in combination with dexamethasone and auto- or allogeneic bone marrow transplantation may also be used. Lesions confined to the skin can be treated with oral retinoids, hydroxychloroquine, interferon alfa, PUVA or UVA-1, cyclosporine, total skin electron beam and Grenz rays therapy (25). Usually, the response to treatment is satisfactory; however, in patients with a chronic disease course of more than three years, achieving complete remission is a severe challenge. As many as 90% of patients with long-term disease fail to achieve this (12).

### 5.3 Buschke’s scleredema

Buschke’s scleredema is one of the fibromucinoses involving the skin. The disease occurs at any age, regardless of race. It affects men more frequently. In 1968, Graff proposed a division into three groups:

· **Type 1** - occurs in children and young adults, with a female predominance. It accounts for about 55% of cases. Onset of the disease is preceded by an infection accompanied by fever - most commonly acute streptococcal pharyngitis, less commonly viral infections. The onset and development of symptoms is dynamic (19, 26). Lesions involve the neck, shoulder rim and trunk (12). Remission usually occurs within 6-24 months.
· **Type 2** - affects approximately 25% of patients, more often women. Most patients have coexisting hematological disorders - primarily paraproteinaemia, but also monoclonal gammopathies (i.e. plasmacytic myeloma, monoclonal gammopathy of undetermined significance) or amyloidosis. It is characterized by an insidious onset and a slow progressive course.
· **Type 3** - involves about 20% of cases, mainly obese men (M:F 10:1). Because it occurs in people with a long history of poorly controlled type 1 and type 2 diabetes treated with insulin it is also called scleredema diabeticorum. The condition affects 2.5-14% of people with diabetes.

Other comorbidities with scleredema include Waldenstorm’s macroglobulinaemia, primary cholangitis, dermatomyositis, ankylosing spondylitis, rheumatoid arthritis, Sjogren’s syndrome, primary hyperparathyroidism, HIV infection and AIDS, IgA deficiency, IgA-related vasculitis and cancer.

The pathogenesis of the disease remains unknown. Fibroblasts, which are activated by a stimulus (including drugs, hyperinsulinism, genetic factors, infections, inflammatory process), are suspected to play a major role. Fibroblasts are responsible for excessive production of type 1 collagen and glycosaminoglycans (19, 26).

In the case of scleredema resulting from diabetes, the cause has been hypothesized to be irreversible collagen glycosylation, activation of fibroblasts for increased collagen and mucin production and their deposition in the dermis. Another hypothesis put forward is the activation of mucin and collagen production as a response to microvascular damage and subsequent hypoxia (27).

The symptoms of the disease change over time. Initially, the skin of the neck is symmetrically affected, followed by the upper limbs and the trunk, sparing the hands and feet. Less frequently, the skin of the face, buttocks and thighs is affected. In the early stages, the skin is pasty, then the above-mentioned areas evolve to a woody induration, taking on an orange peel appearance with hyperpigmentation and erythema. In addition, patients may complain of pruritus, pain and limitation of mobility of the shoulder girdle and problems chewing food.

The course of the disease occasionally involves internal organs - heart, tongue, lungs and pleura, skeletal muscles, liver, spleen, esophagus, parotid glands, eyes. Patients report dry mouth and eyes, swallowing disorders, musculoskeletal pain and symptoms due to pericardial and pleural fluid and hepatosplenomegaly.

The diagnosis is established on the basis of the clinical picture and the result of the histopathological examination. Microscopically, thickened collagen bundles, thickened dermis, clearly demarcated spaces filled with mucopolysaccharides and inflammatory infiltration of vessels by mononuclear inflammatory cells are observed.

Due to the possibility of a coexisting lymphoproliferative process in type 2systemic sclerosis, laboratory tests for paraproteins are recommended.

Differentiating from systemic sclerosis, laboratory tests in patients with scleredema show no antinuclear antibodies and the disease process usually spares the skin appendages. In addition, there is no Raynaud’s phenomenon, no involvement of the distal extremities and the capillaroscopy picture is not characteristic ofsycstemic sclerosis.

The main treatment is UVA1 phototherapy and PUVA. If ineffective, intravenous immunoglobulin is recommended. In clinical practice, bortezomib, methotrexate, cyclosporine, cyclophosphamide, glucocorticosteroids, penicillamine, extracorporeal photopheresis and electron beam radiotherapy have also been administered with a reduction in complaints (19, 26). In recent years, anti-fibrotic treatment based on the use of TGF- β inhibitors has been proposed (12).

Physiotherapy and kinesitherapy are recommended as non-pharmacological treatments. Also, treatment of the underlying cause increases the likelihood of achieving remission. Unfortunately, in people with scleredema types 2 and 3, the disease often progresses slowly despite treatment and can lead to life-threatening complications (19, 26).

### 5.4 Systemic amyloidosis

Amyloidosis is a rare disease caused by the deposition of amyloid fibers in the tissues and extracellular space of organs (28). It can be inherited or acquired. It is also divided on the basis of the areas involved: localized, systemic, ocular, nervous system amyloidosis.

Currently, the classification of the disease is based on the chemical structure of the amyloid. To facilitate diagnosis, the following amyloidosis groups are distinguished:

· AL - primary amyloidosis. Amyloid fibrils are composed of monoclonal immunoglobulin light chains.
· AA - reactive, acquired amyloidosis. A complication of chronic inflammation such as rheumatoid arthritis or chronic infections. The most common systemic amyloidosis. May lead to hepatomegaly, macroglossia, nephropathy and nephrotic syndrome, heart failure with left ventricular hypertrophy, orthostatic hypotension, peripheral and autonomic neuropathy.
· Aβ2M - occurs in people on long-term dialysis therapy.
· ATTR - transthyretin-associated familial amyloidosis. Abnormal amyloid is produced by the liver. It manifests with autonomic and peripheral neuropathy, vitreous opacity and heart failure due to cardiomyopathy (27).

The course of the disease and the clinical picture correlates with the organs involved. The most common are the kidneys (70%) and the heart (60%). Skin involvement occurs in 40% of individuals in the form of hemorrhages around the eyes, waxy thickening of the skin of the fingers, white-yellow papules with associated pruritus on the scalp (19, 28). Sclerosis of the skin of the extremities leads to restriction of joint mobility. Other symptoms reported by patients include fevers and sub-febrile states, general weakness and joint pain (8).

One rare type of amyloidosis is Primary Localized Cutaneous Amyloidosis (PLCA). It predominantly affects people in Asia and Latin America. It involves amyloid deposition in the dermis with sparing of internal organs. Unlike the systemic form, where amyloid fibers are formed from serum proteins and immunoglobulins, in the cutaneous form amyloid is formed from keratin peptides of necrotic keratinocytes. The cause of disease development is unknown. It is suspected that intense scratching may be the cause of amyloid deposition in the skin. A distinction is made between lichenoid (the most common subtype), nodular and macular amyloidosis. The nodular lesions in lichenoid amyloidosis are mainly located on the lower extremities. In macular amyloidosis, the lesions are brown-gray macules visible on the upper part of the squares. The rarest nodular amyloidosis is characterized by hard pink or brown nodules that may cluster into plaques. Lesions are located on the upper and lower extremities (29).

The key to diagnosis is revealing amyloid deposits in the histological specimen using Congo red staining. In the cutaneous form, a biopsy is taken from the affected area, while in the generalized form, subcutaneous adipose tissue is mainly taken. A salivary gland, rectal mucosa or kidney may also be sampled. To classify the type of amyloidosis, immunohistochemistry or tandem mass spectrometry is performed to determine the type of amyloid.

Treatment varies depending on the cause. For primary amyloidosis (with the presence of light chains), the main treatment is bone marrow transplantation. For those not eligible for transplantation, a bortezomib-based chemotherapy regimen is used (28). Clinical improvement after liver transplantation has been observed in patients with ATTR (30). Anti-inflammatory treatment of the primary disease is recommended for secondary amyloidosis (31). In contrast, treatment of primary localized cutaneous amyloidosis is based on the use of high-potency topical corticosteroids and UVA1 phototherapy (29).

### 5.5 Lipodermatosclerosis

Dermato-fat sclerosis is caused by chronic venous insufficiency, limited inflammation of the adipose tissue and subsequent fibrosis of the subcutaneous tissue and skin. It most commonly affects middle-aged and elderly people, and is predominant in women. The lesions are mainly located on the lower extremities, where, due to venous valve insufficiency and venous hypertension, there is increased vascular permeability, hypoxia, activation of the inflammatory process and TGF- β, collagen deposition and release of proteolytic enzymes, and ultimately damage to the skin and subcutaneous tissue. In the acute state, the affected skin resembles a rose - it is vividly red, swollen, excessively warm and tender on palpation (32, 33). With time, it turns a reddish brown color, becomes scaly, and its hardening and atrophy produces the characteristic image of an inverted champagne bottle. Symptoms reported by patients include pain in the affected areas, swelling that increases throughout the day and hard-to-heal ulcers.

Due to the difficulty of wound healing, skin biopsy is rarely performed and the diagnosis is mainly established on the basis of the clinical picture. If a slice is taken, histopathological examination reveals characteristic foci of fat necrosis with calcium deposits in elastin fibers. In the subcutaneous tissue and dermis, hemosiderin deposits - siderosomes - are visible. In recent years, the high usefulness of ultrasonography as a non-invasive diagnostic method and for monitoring the course of the disease and treatment has been explored. However, parameters have still not been defined to speed up the diagnostic and treatment process (34).

The mainstay of treatment is elevation of the lower limbs while sitting or lying down and compression therapy. Pharmacotherapy mainly uses pentoxifylline, hydroxychloroquine, capsaicin. Beneficial effects of anabolic steroids (stanozolol, danazol, oxandrolone) have also been observed, resulting in the activation of fibrinolysis and a reduction in skin induration and pain (32).

## 6 Iatrogenic sclerosis

### 6.1 Drug-induced induration of the skin

Drug-induced scleroderma is a rare and still unexplored condition. There are two types of drug-induced scleroderma: systemic sclerosis and localized scleroderma (morphea). The pathogenesis of the disease is not fully understood. One of the drugs that causes cutaneous sclerosis is bleomycin. In a study in bleomycin-treated patients, increased production of type I procollagen and human lung fibroblasts was noted, which stimulated cells to produce collagen and glycosaminoglycans. In addition, the role of IL-6 as a pro-fibrotic cytokine was taken into account.

The group of drugs that can lead to skin sclerosis is numerous. This includes the previously mentioned bleomycin, L-tryptophan or bromocriptine. Other drugs are listed in **Table 6**.

**Table 6:** Drugs which may induced sleroderma- like skin lesion (35).

• Carbidopa and l-5-hydrozytryptophan • Levodopa • Ethosuximide • Appetite suppressants • Vinyl chloride • Vitamin K1 • Penicillamine • Fosinopril • Triamcinolone • Interferon-alpha • Nivolumab • Pembrolizumab • Pentazocine • Methysergide • Bromocriptine • Ketobemodine • L-tryptophan • Bleomycin • Taxane-based agents • Gemcitabine • Pemetrexed • Uracil-tegafur

The most commonly affected skin areas are the lower limbs, which are initially swollen and successively the skin becomes hard. If the fingers are involved, patients complain of limited mobility. During the course of the disease, ulcer formation may also occur as a result of vascular damage (35). On capillaroscopy, changes characteristic of the active period in Cutolo’s scleroderma can be observed - reduced vascular density, disorganization of the vascular system and numerous megacapillaries. Other symptoms also present in systemic sclerosis are telangiectasias involving the facial skin, microstomia, inability to fully straighten the fingers at the distal interphalangeal joints and complaints due to gastro-oesophageal reflux (8).

Diagnosis is based on a meticulously taken history and tests for systemic scleroderma - antinuclear antibodies, capillaroscopy and assessment of internal organ involvement.

The main differences between drug-induced cutaneous sclerosis and systemic sclerosis are the rare manifestation of Raynaud’s phenomenon, the lack of internal organ involvement and the absence of antinuclear antibodies. Symptoms appear mainly at the age of 50 years, where the onset of scleroderma is mainly observed between the ages of 40 and 60 years. Drug-induced scleroderma occurs with similar frequency in men and women, while systemic sclerosis affects mostly women (90%). The affected area is also a helpful criterion to differentiate the two diseases: in drug-induced scleroderma, the predominant area is the lower limbs, while in systemic sclerosis the distal parts of the limbs are initially affected and with time there is progression to the skin of the proximal parts, i.e. the trunk, arms and thighs. Changes due to vascular damage are rarely observed in drug-induced scleroderma, and if ulcers appear, they do not occur in the fingertips, a typical site for systemic sclerosis.

The most important factor in the differentiation should not be forgotten either - the intake of the disease-inducing drug.

Due to the diverse course of the disease, treatment must be tailored to each individual case. The first step is to discontinue therapy with the suspected drug. In cases where scleroderma leads to limitation of daily activities, steroid therapy may be considered.

In conclusion, due to the possibility of scleroderma occurring during therapy with the drugs in question, the above disease should always be considered during the differential diagnosis of systemic sclerosis (35).

### 6.2 ASIA syndrome

ASIA syndrome is an autoimmune/inflammatory syndrome induced by adjuvants. As the name suggests, the trigger for the syndrome is the activation of the inflammatory/autoimmune process by adjuvants such as silicone implants, drugs, metals, vaccine components or infections. The development of the disease occurs in predisposed individuals - mainly those with the PTPN22 gene, HLA DQ and HLA - DRB1.

The diagnosis is established on the basis of diagnostic criteria:

· Large criteria:
  ▪ Exposure to an external agent (e.g. adjuvants, vaccinations, silicone implants, infections) before the onset of symptoms.
  ▪ Typical clinical picture: muscle pain, muscle weakness, muscle inflammation; joint pain or arthritis; sleep disturbance, fatigue; neurological symptoms; cognitive impairment, memory impairment; fevers, dry mouth.
  ▪ Removal of the causative agent results in a reduction of symptoms.
  ▪ Typical histopathological picture of a specimen taken from the involved organs.
· Small criteria:
  ▪ Presence of autoantibodies or antibodies against adjuvant.
  ▪ Typical HLA (HLA DRB1, HLA DQB1).
  ▪ Development of an autoimmune disease e.g. systemic sclerosis, multiple sclerosis.
  ▪ Other clinical signs, e.g. irritable bowel syndrome.

One example of ASIA syndrome that can mimic scleroderma is silicone implant incompatibility syndrome. The presence of a foreign body such as silicone implants in genetically predisposed individuals can cause stimulation of the immune system, the formation of autoantibodies and, ultimately, the development of an autoimmune disease (36).

The literature reports a case of a patient with breast implants who presented with fatigue, Raynaud’s phenomenon, non-productive chronic cough and dysphagia. Laboratory tests performed revealed the presence of antinuclear antibodies, anti-Scl-70. The patient did not meet the diagnostic criteria for systemicsclerosis. Based on the clinical presentation, the patient was diagnosed with ASIA syndrome (37).

In addition, a recent study by the US Food and Drug Administration (FDA) found high rates of systemicsclerosis, Sjögren’s syndromes and rheumatoid arthritis in people with breast implants, including silicone implants (38).

## 7 Toxic and occupational

Many toxins can cause skin symptoms resembling systemicsclerosis. Examples of compounds are aromatic hydrocarbons, i.e. benzene, toluene, xylene; epoxy resin and white spirit (8).

### 7.1 Vinyl chloride poisoning

Vinyl chloride is a carcinogenic gas used in the production of polyvinyl chloride (PVC). Today, occupational exposure to this compound is rarely observed, although workers exposed to vinyl chloride in the 1970s now present with remote effects of exposure (39).

The clinical and histopathological picture resembles local and systemicsclerosis. Patients present with thickening of the skin of the hands and fingers, Raynaud’s phenomenon, impaired circulation in the vessels of the lower extremities, acroosteolysis of the bones of the distal phalanges of the hands and feet and signs of thrombocytopenia (8, 39). Some patients develop hepatic hemangioma and symptoms of liver failure.

In the differentiation from scleroderma in cases of vinyl chloride poisoning, no antinuclear antibodies are observed in the serum of patients (39), and capillaroscopy shows narrowing of the vascular lumen and subtotal closure of the arterioles (8).

### 7.2 Toxic oil syndrome

In 1981, there was a mass poisoning in Spain after the consumption of refined rapeseed oil, which was sold as olive oil. There were about 351 fatalities and the development of symptoms in about 20 000 people. In 1986, a study was published on a group of people who developed scleroderma-like symptoms. The primary symptoms included swelling and induration of the forearms and lower limbs, muscle pain, contractures in the joints, Raynaud’s phenomenon, ulcerations on the fingertips and eosinophilia observed in laboratory tests. In addition, patients developed lung and esophageal involvement during the course of the disease, causing abnormalities in respiratory mechanics, pulmonary hypertension and dysphagia. Some patients showed renal damage, arthritis, peripheral neuropathy and features of malabsorption syndrome. Features of microvascular damage were observed on capillaroscopy.

Histological preparations showed an inflammatory infiltrate (composed of mononuclear cells and eosinophilia) in the skin, muscle, fascia and vascular wall, fibrosis of the dermis, thickening of the vascular inner membrane and atrophy of the appendages (40).

Currently, patients continue to experience the effects of poisoning, which negatively affect their daily lives (41).

## 8 Metabolic

### 8.1 Hypothyroidism and hyperthyroidism

Myxedema is one of the cutaneous manifestations of severe thyroid hormone imbalance. It is more common in thyroxine and triiodothyronine deficiency, but can also affect patients with hyperthyroidism (12). The skin lesion is characterized by swelling without leaving an indentation when pressed with a finger (42). It can be localized on the eyelids, hands, feet, in the pre-shin area or involve the whole body.

Histology shows thickening of the dermis with deposits of glycosaminoglycans, chondroitin sulfate present. The main treatment is thyroxine replacement therapy (12).

Thyroid dermatopathy, also known as fore-shoulder oedema, occurs mainly in Graves-Basedow disease, although it has also been observed in patients with Hashimoto’s disease. The clinical picture presents a sharply demarcated, hard swelling accompanied by nodules or papules covering mainly the pre-shin and dorsal regions of the feet.

In Graves’ Basedow disease, treatment is based on the use of thiamazole preparations, possibly radioactive iodine or surgical treatment (42).

### 8.2 Cutaneous porphyria late onset

Cutaneous late-onset porphyria (PCT) belongs to metabolic diseases and is the most common type of porphyria. It occurs in 1 in 10 000 people. It mainly affects middle-aged people. Women and men are affected with equal frequency.

The cause of the disease is a dysfunction or deficiency of uroporphyrinogen decarboxylase (UROD), which is responsible for the conversion of uroporphyrinogen III into coproporphyrinogen III, leading to the accumulation of porphyrins in the body - mainly in the skin and liver.

Risk factors for symptoms in patients with PCT include alcohol consumption (80-90% of patients), smoking (81% of patients), haemochromatosis (53% of patients), HCV, HIV infection (13% of patients), taking estrogen preparations (66% of patients), and iron supplementation.

There are three types of cutaneous porphyria of late onset:

· **Type 1** - the most common (80%), caused by the presence of an inhibitor of the enzyme uroporphyrinogen decarboxylase. The production of the inhibitor is provoked by the presence of oxygen free radicals resulting from iron overload.
· **Type 2** - 20% of cases, autosomal dominantly inherited
· **Type 3** - rare, familial occurrence, unknown genetic mechanism

The disease can be asymptomatic. Unlike other porphyrias, patients present only with skin symptoms. After exposure to sunlight, blisters appear on the skin, which heal leaving discolouration, scarring and yellowish-white papules. The lesions mainly occur on the back of the hands and forearms, face and ears. In addition, there is hardening of the skin, delayed wound healing and contractures. When the scalp is involved, diffuse scarring alopecia is observed. Some women present with excessive hair on the temples, cheeks and forearms,

The porphyrins present in the body, when exposed to light (mainly at 410 nm), release energy that causes the formation of ‘singlet-excited oxygen’. This leads to peroxidation of cell membrane lipids, disruption of cell stability and cell death. The activation of complement cells, mast cells and TGF-β, which starts the process of fibrosis and causes scleroderma-like skin sclerosis, is also responsible for the skin symptoms.

The diagnostic work-up includes assessment of serum and urine porphyrin concentrations. It should be noted that in this disease entity, the concentration of δ-aminolevulinic acid (ALA) in the excreted urine is normal. Skin biopsy is not obligatory. The histological picture of the specimen taken from the sclerosed area looks like that in scleroderma. Porphyrin deposits and subepidermal blisters are characteristic features.

The basis of treatment is patient education and avoidance of porphyrogenic factors - sunlight, alcohol, cigarettes and others. For patients found to have excessive iron accumulation in the body, bloodletting is the recommended treatment - the treatment goal is a ferritin concentration <20 ng/ml. Chloroquine and its derivative, hydroxychloroquine, are also used in therapy. In case of contraindications to bloodletting and antimalarials, the chelating drugs deferoxamine or deferasirox can be used.

Prognosis is influenced by exposure to risk factors. Patients who do not comply with medical advice have a higher risk of recurrence. Monitoring of the disease involves annual monitoring of serum and urine porphyrin levels, as an increase precedes the onset of clinical symptoms. Complications of the disease besides skin lesions include hepatic steatosis, increased risk of cirrhosis and hepatocellular carcinoma (43).

### 8.3 Phenylketonuria

Phenylketonuria is a genetically determined metabolic disease. It is inherited in an autosomal recessive manner. In the US population, it occurs in 1 in 15 000, more commonly in Caucasians and Native Americans. The disease is caused by a mutation in the gene encoding phenylalanine hydroxylase. This leads to a disruption in the conversion of phenylalanine to tyrosine, resulting in high serum phenylalanine and low serum tyrosine concentrations. Excess phenylalanine and its metabolites cause damage to the nervous system, while tyrosine deficiency leads to decreased production of dopamine, adrenaline and noradrenaline and decreased melanin synthesis. Note that high phenylalanine concentrations can also result from tetrahydrobiopterin (BH4) deficiency.

Initially, neonates do not manifest the disease. After a few months of life, the first symptoms appear, i.e. persistent vomiting, mousey skin and urine odor, skin lesions in the form of eczema, pale skin and hair, microcephaly, neurological signs - convulsions, hyperactivity.

In the absence of treatment, patients develop sclerosis of the skin of the proximal parts of the limbs, with sparing of the hands and feet, which occurs when a restrictive phenylalanine-restricted diet is initiated (8).

There is now a screening programme that measures the ratio of phenylalanine to tyrosine in a newborn’s blood spot using tandem mass spectrometry (MS/MS). This allows early diagnosis of the disease, prior to manifestation of symptoms and structural damage.

Treatment consists of a low phenylalanine diet, and there is an enzyme substitution therapy, PEGylated phenylalanine ammonium lyase (pegvalase), which can be used in adult patients.

Recent studies have shown that cognitive impairment occurs in some cases despite early treatment initiation (44).

### 8.4 Diabetic cheiroarthropathy

Diabetic cheiroarthropathy, also known as restricted joint mobility syndrome, is a complication of long-term, poorly controlled type 1 as well as type 2 diabetes (11). Men and women are affected with equal frequency (7). The disease is caused by non-enzymatic glycosylation of collagen and activation of fibroblasts to overproduce collagen and other matrix proteins due to long-term hyperglycemia.

The main symptoms are induration and thickening of the skin, involving the hands with sparing of the palms. The skin involvement leads to reduced mobility of the metacarpophalangeal and interphalangeal joints of the hands resulting in an inability to fully extend the fingers and the occurrence of a ‘prayer sign’. In the course of the disease, extension of the affected area to the distal parts of the upper and lower limbs may also occur.

During the diagnostic process, a skin biopsy is taken of part of the tendon - histopathological examination reveals skin fibrosis, sclerosis of the tendon sheaths (11), deposition of excess collagen and mucin (7). In addition, imaging studies such as ultrasound and MRI show thickening of the subcutaneous tissue and flexor tendon sheaths.

Important facts in the differentiation from scleroderma are the absence of telangiectasia, the absence of Raynaud’s phenomenon and a normal capillaroscopy picture and the absence of antinuclear antibodies (7, 11).

The cornerstone of treatment is rehabilitation and maintenance of normal glycaemic control, which can prevent disease progression (11).

## 9 Paraneoplastic syndromes

The appearance of Raynaud’s phenomenon, sudden onset and dynamic development of skin sclerosis, sclerodactyly, absence of capillaroscopy changes, absence of antinuclear antibodies and absence of gastrointestinal symptoms present in systemic sclerosisshould increase oncological alertness. The above-mentioned symptoms may occur in the course of hematopoietic proliferative process, neuroendocrine tumors and solid tumors (19). Activation of fibroblasts by TGF- β is responsible for the fibrotic process in cancer (7). On histological examination, the picture may be indistinguishable from systemic sclerosis (19).

### 9.1 Carcinoid syndrome

It is a syndrome caused by the secretion of hormones and mediators, i.e. serotonin, neurokinin A, substance P, by neuroendocrine tumors. Symptoms include: attacks of redness of the face and neck, diarrhea, right heart fibrosis, hypotension, tachycardia, bronchospasm and skin lesions, i.e. telangiectasias and induration of the skin. The differences between the skin involvement in the course of carcinoid syndrome and in the course of scleroderma are the absence of Raynaud’s phenomenon, initial involvement of the skin of the lower extremities, successively of the upper extremities, and sparing of the distal parts of the fingers (8).

### 9.2 Neoplastic hypereosinophilic syndrome

Hypereosinophilic syndrome is characterized by peripheral blood eosinophilia >1500/µl with associated organ damage. More than half of the patients present with skin manifestations, i.e. urticaria, livedo, angioedema, erythroderma, erythema annulare centrifugum, erythematous papules, splinter hemorrhages, acral necrosis, plaques, nodules, palpable purpura, Wells syndrome, pruritus. Changes typical of scleroderma may also be present. These include swelling progressing to induration of the skin and subcutaneous tissue of the upper and lower extremities. Histopathological examination shows inflammatory infiltration of the subcutaneous tissue composed of eosinophilia and lymphocytes, thickening of the stratum corneum (8).

## 10 Hereditary sclerosis

Hereditary diseases with skin sclerosis include acrogeria, Werner’s syndrome, stiff skin syndrome, Huriez syndrome, Winchester syndrome, GEMSS syndrome, pachydermoperiostosis, among others. Below are descriptions of selected disease entities.

### 10.1 Werner syndrome

Werner syndrome is one of the progeria syndromes, inherited autosomal recessively. The disease affects 1 in 100 000 people worldwide. The highest number of cases has been recorded in Japan (1 in 20 000/40 000 births).

The cause of the disease is a mutation of the WRN gene, which is responsible for normal replication, maintenance of telomere stability and DNA repair. Its damage results in symptoms of premature aging at the end of adolescence or in the second decade of life. Patients develop atrophy of the skin, adipose tissue and sarcopenia. The skin - especially of the distal parts of the extremities - is hard with ulcerations, the face takes on a bird-like appearance, making the disease picture reminiscent of systemicsclerosis. Other symptoms include a squeaky voice, hair loss, cataracts, soft tissue calcinosis (mainly of the Achilles tendons), low BMI, osteoporosis (mainly of the distal limb bones). In addition, metabolic disorders and their complications develop - type 2 diabetes, hyperlipidaemia, atherosclerosis, hypertension, myocardial damage, stroke. Approximately 10% of patients develop tumors - mainly soft tissue sarcomas (including schwannoma, osteosarcoma, leiomyosarcoma) (45).

Genetic testing is used in diagnosis, also prenatal testing in high-risk individuals. Histopathological examination of a skin section shows features of fibrosis and the presence of collagen fibers in place of fatty subcutaneous tissue (8). Patients with a confirmed diagnosis are monitored for the development of metabolic disorders, soft tissue tumors, breast or colorectal cancer.

There is currently no causal treatment. The only treatment is to alleviate symptoms and delay organ damage. Life expectancy is 50 years and the cause of death is usually cancer and cardiovascular disease (45).

### 10.2 Stiff skin syndrome

Stiff skin syndrome is a rare genetic syndrome, inherited autosomal dominantly, caused by a disruption in the sequence of the FBN1 gene, responsible for the normal structure of the fibrillin 1 protein, or in the case of a localized form and in some cases a diffuse form, mosaic activation of fibrosis-promoting genes may be the cause. The clinical picture consists of localized or diffuse thickening and hardening of the skin, which can lead to restricted joint and thoracic mobility, contractures, gait disturbances and postural defects. The skin may show excessive hairiness, hyperpigmentation, erythema or wrinkling. The most commonly affected areas are the shoulder rim, lower back and thighs (46). Symptoms usually occur already in newborns or in children at a young age (47). In the limited form, the first manifestation of the disease occurs later and the course is milder than in the disseminated type (46).

Histological examination of the skin biopsy shows mucin deposits, thickened collagen bundles, adipocyte entrapment and also mucopolysaccharide deposits can be visualized in the connective tissue by Alcian blue staining. No inflammatory process is found in the biopsy (46, 47). Furthermore, in contrast to systemic sclerosisand morphea, immunohistochemistry examination shows CD34 expression. Diagnosis is based on the correlation of symptoms and histopathological findings; genetic testing is not a diagnostic criterion.

The main treatment is rehabilitation care. Drugs used include mycophenolate mofetil, secukinumab and losartan (46).

### 10.3 The Huriez syndrome

A rare autosomal dominant syndrome consisting of scleroatrophic of the distal parts of the limbs, underdevelopment of the nails and keratosis of the skin of the palms and soles. In addition, the disease leads to the development of squamous cell carcinoma (SCC) in approximately 15% of individuals. To date, the gene responsible for the disease has not been discovered. Research to date has put forward the suspicion that a mutation in the gene encoding the p53 protein results in the activation of the neoplastic process.

Treatment is based on the topical use of keratolytics, emollients and retinoids (the latter also in oral form).

Insystemic slcerosis, familial incidence is less common and cutaneous keratosis is rare (48).

## 11 Summary

Scleroderma-like syndromes form an extensive group of rare conditions that share the common feature of systemic sclerosis.The main features that distinguish scleroderma-like syndromes from systemic sclerosisare the absence of Raynaud’s phenomenon, the absence of ANA and the absence of lesions or an uncharacteristic picture on capillaroscopy. It is important during the differential diagnosis to know the causative factors and the characteristic features in the clinical and histopathological picture of each disease. It is hoped that the information presented in this article will facilitate the diagnosis of systemic sclerosisand scleroderma-like syndromes and allow faster initiation of therapy.

The study “Scleroderma mimics in cohort from an EUSTAR centre” serves aimed to assess the types of scleroderma mimics encountered in a tertiary care center and highlight the difficulties in diagnosis (49).

The researchers evaluated a cohort of 140 patients admitted to their clinic with suspected SSc between January 2007 and December 2017. Of the 140 patients evaluated, 10 (7.14%) were found to have scleroderma mimics. These patients had initially presented with symptoms suggestive of SSc, including severe RP, acroosteolysis, or symmetric skin thickening. However, upon detailed evaluation, they did not demonstrate typical features of SSc such as organ involvement, abnormal capillaroscopic patterns, or positive scleroderma-specific antibodies. The scleroderma mimics identified included conditions such as primary RP, Hajdu-Cheney syndrome, solvent-induced scleroderma, scleroedema adultorum, scleromixedema, and eosinophilic fasciitis. Each mimic presented with distinct clinical features and diagnostic challenges. For instance, patients with solvent-induced scleroderma showed complete resolution of skin sclerosis after eliminating solvent exposure, while those with scleroedema adultorum did not have underlying gammopathy or infections. Patients with eosinophilic fasciitis exhibited extended skin thickening with eosinophilia and responded partially to immunosuppression. The study underscores the importance of considering scleroderma mimics in the differential diagnosis of SSc, particularly in cases where clinical features are atypical or discordant with typical SSc findings. It highlights the need for a comprehensive diagnostic approach, including clinical, laboratory, and imaging assessments, to accurately differentiate between SSc and its mimics and prevent diagnostic delays (49).

In summary all rheumatologist have a critical diagnostic challenge encountered in patients with scleroderma, highlighting the complexity of distinguishing systemic sclerosis from conditions mimicking its clinical presentation. By addressing the difficulties in accurately diagnosing scleroderma patients, our study underscores the pressing need for improved diagnostic strategies and heightened clinical awareness to ensure timely and appropriate management.

## Author contributions

KR: Conceptualization, Funding acquisition, Investigation, Supervision, Visualization, Writing – original draft, Writing – review & editing. MD: Formal analysis, Methodology, Visualization, Writing – original draft, Writing – review & editing. AL: Formal analysis, Project administration, Supervision, Visualization, Writing – review & editing. AR: Visualization, Writing – original draft. MO: Formal analysis, Supervision, Validation, Writing – review & editing.
