# Corrigendum: Scleroderma and Scleroderma-like syndromes

**DOI:** 10.3389/fimmu.2024.1456067

**Published:** 2024-07-22

**Authors:** Katarzyna Romanowska-Próchnicka, Martyna Dziewit, Aleksandra Lesiak, Adam Reich, Marzena Olesińska

**Affiliations:** ^1^ Department of Connective Tissue Diseases, National Institute of Geriatrics, Rheumatology and Rehabilitation, Warsaw, Poland; ^2^ Department of Dermatology, Pediatric Dermatology and Dermatological Oncology, Medical University of Lodz, Lodz, Poland; ^3^ Department of Dermatology, Institute of Medical Sciences, Medical College of Rzeszów University, Rzeszów, Poland

**Keywords:** systemic sclerosis, morphea, scleroderma, scleroderma-like syndromes, syndromes of inflammatory/autoimmune, genetic, toxic

In the published article, there was an error in affiliation 3. Instead of “Department of Dermatology, Pediatric Dermatology and Dermatological Oncology, Medical University of Lodz, Lodz, Poland”, it should be “Department of Dermatology, Institute of Medical Sciences, Medical College of Rzeszów University, Rzeszów, Poland”.

In the published article, there was an error regarding the affiliations for authors Katarzyna Romanowska-Próchnicka, Martyna Dziewit and Marzena Olesińska. Instead of having both affiliations 1 and 2, they should only have affiliation 1.

In the published article, there was an error regarding the affiliation for author Aleksandra Lesiak. Instead of having affiliation 3, they should have affiliation 2.

The authors apologize for these errors and state that they do not change the scientific conclusions of the article in any way. The original article has been updated.

